# Impact of some heavy metal accumulation in different organs on fish quality from Bardawil Lake and human health risks assessment

**DOI:** 10.1186/s12932-023-00084-2

**Published:** 2024-01-11

**Authors:** Ghada Y. Zaghloul, Hoda A. Eissa, Amira Y. Zaghloul, Mahmoud S. Kelany, Mohamed A. Hamed, Khalid M. El Moselhy

**Affiliations:** 1 Marine Chemistry Lab National Institute of Oceanography and Fisheries, Cairo, Egypt; 2 Fish Reproduction and Spawning Lab National, InstituteofOceanographyand Fisheries, Cairo, Egypt; 3 Senior Specialist Egyptian Holding Company for Biological Products and Vaccines, VACSERA, Cairo, Egypt; 4 Microbiology Lab National Institute of Oceanography and Fisheries, Cairo, Egypt; 5 Marine Pollution Lab National, Institute of Oceanography and Fisheries, Cairo, Egypt

**Keywords:** Bio-chemical composition, Heavy metals, Gills, Liver, Muscle, Fish, Risk assessment, THQ, CSR, Bradaiwl Lake

## Abstract

Bardawil Lake is a unique aquatic ecosystem that provides a habitat for various fish and other marine organisms. This study aimed to analyze the quality of fish species to prove that this lake is free of pollution, not other Egyptian lakes, due to the accumulation of some heavy metals (Cd, Pb, Cu, and Zn) in various tissues of fish species that were caught from this lake. Thirty-five fish samples were caught during the Spring of 2018 from seven different species: *Mugil cephalus, Liza auratus, Sparus aurata, Dicentrarchus labrax, Siganus rivulatus, Anguilla angilla, and Solae solea*. The Association of Official Analytical Chemists methods using a spectrophotometer determined the biochemical composition. In contrast, atomic absorption spectrometry (AAS) was employed to determine the heavy metals expressed by µg/g wet weight. Results exposed that the accumulation of essential micronutrient (Cu, Zn) content was higher than toxic elements (Cd & Pb) in muscles in order to Zn > Cu > Pb > Cd. Muscles < gills < liver in order of all metals except Pb with order muscles < liver < gills. The metals studied in the muscles were lower than those set by the WHO and the EU standards. The carcinogenic risk with lower allowable limits of 1 × 10^–6^ to 1 × 10^–4^ in both normal and high consumption groups; target and total target hazard quotients (THQ & HI) in muscles were < 1. The biochemical composition level was highest in the liver, except for protein, which was highest in muscle for all fish species. There is no evidence of harmful contaminants in the muscular tissue of the fish sampled from Bardawil Lake, although fishing activity. However, customers should know that health concerns may be associated with overeating fish.

## Introduction

Fish and their products play a crucial role in many aspects of a healthy human diet, particularly for individuals who avoid red meat, are immune-compromised, malnourished, pregnant, or nursing (Obeka et al., 2020; [43]. Fish is an appreciated digestible protein source with fats, fat-soluble, essential amino acids, vitamins, trace elements, and long-chain omega-3 polyunsaturated fatty acids [43]. Additionally, incorporating fish into one's diet can help prevent cancer, heart disease, high blood pressure, Alzheimer's, and inflammatory diseases [72]. Factors such as species, reproductive cycle, age, sexual development, dietary area, sex, climate, season, and muscle type all play a role in the biochemical composition of fish muscles, which can vary greatly [9, 21]. The nutrient worth of different fish types changes with the seasons and is not constant. Due to the quantity, diversity, and purity of its component amino acids, fish is an excellent protein supply [9]. Pollution bio-monitoring uses various fish organs, with the liver being a crucial organ for heavy metal accumulation in fish metabolism [63, 74]. The muscle is essential to human nutrition and is an excellent instrument for health risk evaluation in heavy metal contamination. At the same time, the gills have an enormous surface area exposed to water and receive the proper quantity of metal ions [56]. Deficits or excesses of elements like zinc (Zn) and copper (Cu) can harm human health, but they are essential for the growth of specific biochemical processes of biological systems. Nonessential metals, such as lead (Pb) and cadmium (Cd), have no biological purpose and may cause cancer. Due to their high bioaccumulation rate, Pb and Cd are considered the most toxic heavy metals for aquatic organisms [24].

The ingestion of fish can pose a risk to human health and aquatic ecosystems due to heavy metal pollutants [11, 30]. So, trace element level analysis in fish is essential [18, 25, 58, 73]. Fish species can also be bio-indicators of environmental pollutants, with metals accumulating in their tissues, particularly in the muscles [14, 18, 40]. Additionally, variables such as climate, time of year, fish species, and developmental stage may all affect the concentrations of trace elements in their flesh (Łuczyńska et al., 2019; [32], and excessive accumulation of trace elements can lead to harmful health effects [45]. Gills accumulate significant amounts of metal ions with their large surface area exposed to water [56]. Elements like zinc (Zn) and copper (Cu) are essential and vital for enzymatic and physiological functions and specific biochemical processes in biological systems. However, deficiencies or excesses can harm human health. The human body contains less than 0.01% zinc (Zn) and copper (Cu), and the recommended daily allowance (RDA) is often less than 100 mg/day [43]. Excessive (Cu) and (Zn) intake can lead to health complications such as kidney and liver damage [28]. On the contrary, nonessential and toxic metals like lead (Pb) and cadmium (Cd) have no biological purpose and can cause cancer. Long-term exposure also has a toxic impact on the kidneys, namely on the numerous enzymes involved in protein reabsorption in the renal tubules, ultimately leading to kidney failure. Accumulating trace elements in organisms above what is required for metabolic activities might negatively affect health [45].

Risk assessment is a valuable technique used to evaluate potential impacts of pollutants. In the case of heavy metals, health risk assessments are commonly conducted to assess the overall exposure of individuals to these contaminants in a particular location [35, 53, 69]. The potential carcinogenic or non-carcinogenic effects of contaminants on humans are usually considered during risk assessments. Unfortunately, sufficient research is not evaluating the combination of heavy metals' ecological risk and biochemical composition from fish in Bardawil Lake [16]. In construction, physical and chemical parameters for Bardawil Lake locations (water quality) were assessed or investigated in the area as in the previous study [75].

Bardawil Lake plays a significant role in Egypt's lake fisheries industry as it has low contamination levels, and most of its catch is exported. This shallow hyper-saline lagoon extends approximately 90 km in length and up to 22 km in width, with a depth range of 0.3 to 5 m. It covers an area of 650 km^2^ and is located along Sinai's Mediterranean coast, separated from the sea by a sandbar of 100 m to 1 km in width. The lagoon is connected to the sea at its easternmost point by two natural inlets (Bughaz Zaranik and Abo Salah) and two manufactured waterways (Boughaz I&II). It spans from 31°03 00 to 31°14 00 N latitude and from 32°41 00 to 33°30 00 E longitudes, with a maximum width of 14 km at its widest point. The lagoon hosts various ecosystems, including saline sand flats, hummocks (neck as), open water, wet salt marshes, stable dunes, inter-dune depressions, and movable dunes, collectively representing six different habitat types. Salt production and fishing are the primary economic activities in the lagoon [17].

This study aimed to analyze with follow-up the accumulation of some heavy metals (Cd, Pb, Cu, and Zn) in various tissues (gills, liver, and muscles) from economic fish species *(Mugil cephalus, Liza aurata, Sparus aurata, Dicentrarchus labrax, Siganus rivulatus, Anguilla anguilla, and Solae solea)* was caught from this vital lake. It was also projected that, like the other Egyptian lakes, this one was negatively impacted by the health risks posed to the general and fishing populations due to fish intake. Furthermore, understanding the effects of environmental conditions and changing organic on fish tissue composition is crucial for ecologists to establish and maintain a suitable dam water environment for producing high-quality fish generations.

## Material and methods

### Studying area

**Fig. 1:**
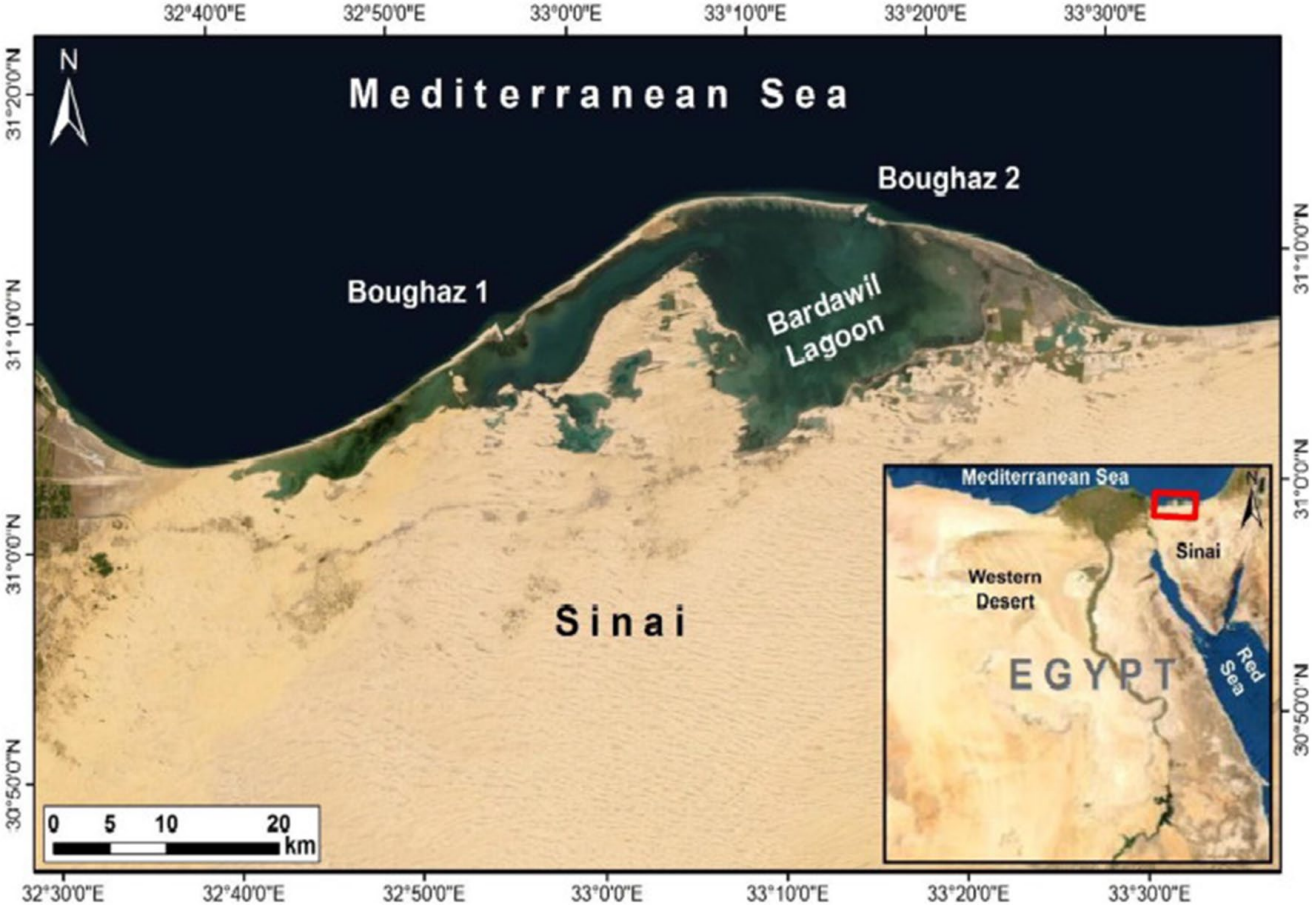
Studying area (Bardawil Lake) during Spring, 2018

One of Egypt's five northern lakes, Lake Bardawil, is one of the most important Egyptian lakes wetlands on the Sinai Peninsula's northern shore near the Mediterranean Sea and is free of pollution, whether agricultural or industrial. The fish caught from the lake are of high quality (Fig. 1). Located between 32°40′E and 33°30′E and 31°03′N and 31°14′N. The average depth of the pond is 1.21 m, the highest depth is 6.5 m, and the lowest depth is 0.3 m [47]. It is about 85 km long and is divided from the Mediterranean by a beach anywhere from 100 m to 1 km wide. The water can enter the lagoon through one of two constructed channels (Boughaz I and II) on the western side or through a natural channel (El-Zaranik) on the eastern side (Bek et al., 2019).

Open Ocean, moist salt wetlands, salty sand plains and hummocks (neck as), stabilized dunes, inter-dune depressions, and movable dunes are just some of the six ecosystem types in Bardawil Lagoon. It is home to the Al Zaranik region and is a crucial stopover for migratory wildlife. The primary commercial operations in the estuary, home to various habitats, are fishing and salt manufacturing. Three inlets lead to the sea from this lagoon, making it the least contaminated of the Mediterranean's coastal lagoons [17].

### Methods for Sampling and Preparing Samples

In the Spring of 2018, 35 individual fish belonging to seven species (*n* = 5) *(Mugil cephalus, Liza auratus, Sparus aurata, Dicentrarchus labrax, Siganus rivulatus, Anguilla anguilla, Solae solea)* that are commonly consumed and were collected from fishermen in Bardawil Lake. Upon collection, the samples were placed in plastic containers before being shipped in an ice chest to the National Institute of Oceanography and Fisheries (NIOF) facility in Suez, Egypt. Morphometric analyses were performed in the lab (total length and weight), ecological and biological information was recorded for each fish species, as summarized in (Table 1), and finally, all samples were analyzed in triplicate.

**Table 1:** The ecological characteristics, feeding habits, and morphometric measurements of the studied fish species in Bardawil Lake, Egypt

Fish name		Living habitat		Feeding Habitat		Weight (g)	Length (cm)
Scientific	Common						
*Mugil Cephalus* Linnaeus, 1758	Flathead grey mullet	Bentho-pelagic	Marine; freshwater; brackish	Carnivores	Feeding on detritus, micro-algae, and benthic organisms (catadromous)	80.58 ± 45.12	18.05 ± 9.83
*Liza auratus*	Golden grey mullet	Pelagic-neritic	Marine; freshwater; brackish; pelagic-neritic, catadromous		Feed on small benthic organisms, detritus, and occasionally on insects and plankton (catadromous)	177 ± 30.52	23.75 ± 13.79
*Sparus Aurata*	Gilthead sea bream	Demersal	Marine; brackish		Feed on shellfish, including mussels and oysters	334.00 ± 146.89	22.75 ± 8.03
*Dicentrarchus Labrax*	European seabass		Marine; freshwater; brackish		Feed chiefly on shrimps and mollusks, also on fishes	231.51 ± 101.50	25.80 ± 13.01
*Siganus Rivulatus*	Rabbitfish	Pelagic	It lives in rocky and sandy bottoms	Herbivorous	Feeds on algae seeds mainly by grazing on algae	128.91 ± 36.40	21.53 ± 2.22
*Anguilla Anguilla*	European eel	Demersal	Marine; freshwater; brackish; catadromous	Carnivores	Its food includes virtually the whole aquatic fauna	27.10 ± 5.83	65.53 ± 41.04
*Solea Solea* (Linnaeus, 1758)	Common sole		Marine; brackish; oceanodromous		Adults feed on worms, mollusks, and small crustaceans at night	119.95 ± 70.26	20.65 ± 12.94

### Heavy metal analysis

The (UNEP/IOC/IAEA/FAO, 1990) [37] was followed to separate and carefully weigh the organs, which were then digested using Conc. HNO_3_ in Teflon tubes. When the first puff of golden smoke vanished, digestion was deemed complete. After condensing the contents using double-distilled water to 10 mL, the mixture was filtered and transferred to a clean screw-capped plastic container for cooling. Before analysis was needed, the receptacle was labeled and stored at 4^◦^C.

The APDC/MIBK extraction method described in (APHA, 2012) was utilized in conjunction with an (AAS) atomic absorption spectrophotometer (Perkin-Elmer Model Analyst 100) to determine heavy metal (Cd, Pb, Cu, and Zn) concentrations. The findings were represented as µg/g of sampled material. According to [67], the total amount of metal found in the fish samples was compared using the Metal Pollution Index (MPI), which was determined by multiplying the measured heavy metal concentrations and then taking the fourth root of the result (Eq. 1)

1 $$MPI\, = \,\left( {M_{cd} \, \times \,M_{Pb} \,\, \times \,M_{Cu} \,\, \times \,\,M_{Zn} } \right)^{{\left( {1/n} \right)}}$$

MPI is the metal pollution index, M is the concentration of metals measured and expressed (µg/g), and n is the number of metals measured.

#### Quality control

The analytical grade purity of the chemicals utilized and the processing of reagent blanks with each batch of samples guaranteed precision. De-ionized distilled water was used in all aqueous solutions, and 10% nitric acid was let to soak in all glass and plastic containers overnight before being cleaned. Quality control samples fared well when measured against acid blanks, with heavy metal readings within the allowed range and a metal recovery rate of 90.4% to 97.5%.

Metal nitrate solutions are the gold standard for measuring metal concentrations. These reference materials are BDH-grade pure and contain a concentration of 1000 ppm. The best wavelength (228.8, 283.3, 213.9, and 324.8 nm) and slit width (0.7 nm) for Cd, Pb, Cu, and Zn AAS determinations, respectively. The detection limits for efficient atomic spectroscopy methods ranged from 1 to 100 ppb when using basic standards in a dilute aqueous solution. Cd and Pb have detection limits of 0.8 and 30 ppb, respectively, whereas Cu and Zn have detection limits of 1.5 ppb each. All detection thresholds are based on a three-standard deviation (98% confidence) interval. An accuracy between 8.5 and 18.0% was considered acceptable for these metals.

### Evaluation of potential human health

Human health risk assessment is all about assessing the risks that carcinogenic and non-carcinogenic chemicals pose to human health. The risk assessment procedure consists of four steps: measuring exposure, calculating toxicity (dose–response), identifying hazards, and describing risks [65]. The risks associated with consuming contaminated fish have been evaluated in various approaches by different authors [7, 8] a,b,[1, 74].

#### Non-carcinogenic hazard (THQ)

Non-cancer risks to human health from exposure to heavy metals are quantified using the target hazard quotient. The ratio of the exposure dosage to the reference dose (RfD) is a valuable tool for assessing the dangers of metal pollution [36, 71] (Table 2). According to USEPA [66], the THQ concentration was calculated using the following formula (2):

2 $$THQ = { }\frac{{Efx{ }EDxFDCx{ }Cm}}{{RfDx{ }BWxTA}}{ } \times \,10^{ - 3}$$

**Table 2:** Exposure parameters used for the health risk estimations through consumption of fish [65]

Parameters	Values	
	Unit	Adult
Body weight (BW.)	Kg	70
Exposure frequency (EF.)	Days/year	365
Exposure Duration (ED.)	Years	70
Ingestion Rate of Fish (FIR) [74]	g/day	64.0 g/day for a normal consumer 200.0 g/day for a higher consumer
Average Time	Days/year	
For non-Carcinogenic	365 × E_D_	
Reference dose mg/kg/day (RfD) [74]	Cd = 1 × 10^–3^, Pb = 4 × 10^–3^, Cu = 4 × 10^–2^, Zn = 3 × 10^–1^	
Cancer Slop	Cd = 0.38 and Pb = 8.5 × 10^–3^	

FDC: The average daily food intake of fish muscle (g/person/day) was between 64 and 200 g/day for normal and high consumers [21], FAO/WHO, 2015), where Ef and ED are the number of times exposed and the number of years exposed, respectively. Cm is the concentration of heavy metals in the studied sample (µg/g.wet/wt), TA is the average exposure duration in years, BW is the average weight of an adult in Egypt, and finally, RfD. is the reference dose Table (2) lists the exposure criteria used in the US Environmental Protection Agency's (2015) risk assessment of fish intake. Eating fish is advantageous to health when the THQ is < (1) [66], while eating fish with a THQ > 1 is riskier.

#### Cancer risk assessment

Cancer slope factor (CSF) was used to convert the ADD of the heavy metal over a lifetime of exposure to the risk of an individual developing cancer [65] as part of a carcinogenic risk assessment that estimates a person developing cancer over a lifetime due to exposure to the potential carcinogen. The incidence of cancer was estimated using Eq. (3).

3 $${\text{Cancer Risk}}\, = \,\frac{{Efrx{ }EDxFiRx\,C}}{{{ }BWxTA}}\, \times \,10^{ - 3} \,\, \times \,{\text{CSF}}$$

CSF is the carcinogenic slope factor set by USEPA [65] (Table 2).

#### Hazardous Risk (HI)

The total hazard quotient, or the hazard index (HI), is the summation of the target hazard quotients (THQs) for all the heavy metals analyzed in a particular species. The HI estimates the cumulative risk associated with exposure to multiple heavy metals [66]. HI was calculated by adding the THQ values for each heavy metal in each species using the following formula (Eq. 4) [41] and [2]

4 $${HI=THQ}_{Cd}+{THQ}_{Pb}+{THQ}_{Cu}+{THQ}_{Zn}$$

THQ is below 1, indicating no threat to human health [66]. However, if the THQ is less than 1, there may be a danger to health, and appropriate precautions should be taken. When the HI > 1, there may be a concern for potential health risks [57].

### Biochemical composition analysis:

Each studied fish species was sampled in triplicates, and liver and muscle tissues were isolated and preserved on an ice plate before being frozen at −20 °C for more biochemical composition analysis. The samples were homogenized at 0 °C with 0.1 M phosphate buffer (pH 7.4) using an electric homogenizer (Wise stir Hs-30E, Germany) and then centrifuged at 4000 rpm for 15 min in a cooling centrifuge (SIGMA, Germany). The resulting supernatant was stored at −20 °C for later analysis. The analytical quality of all reagents utilized. (MERC, Germany). Association of Official Analytical Chemists (AOAC, 2016) used conventional techniques to identify the biochemical composition of fish muscles and liver.

#### Moisture content measurements

The method specified by [34] was followed to determine the moisture content of the fish samples. First, three homogenized replicates of the samples were weighed and transferred to reweighed aluminum dishes. Subsequently, the samples were dried in a hot air oven at 105 °C until a constant weight was attained. The samples were then cooled to room temperature in a desiccator, and the difference between the wet and dry weights was calculated as the water percentage in (Eq. 5), expressed as a percentage (%):

5 $$\% {\text{ Moisture}} = ({\text{weight of the sample before drying}}\, - \,{\text{the weight of the sample after drying}})\, \times \,100$$

#### Crude protein measurements

The crude protein content in the fish samples was estimated using the Kjeldahl method, with 6.25 as the conversion factor to convert the total nitrogen after acid digestion into crude protein. In a Kjeldahl beaker, two games of homogenized fish, ten games of catalyst, 25 ml of pure H_2_SO_4_, and three glass beads were measured. First, powdered potassium sulfate, copper sulfate, and selenium were combined in the following ratio: 94.8:5:0.2 and used to process the contents until transparent. Next, the mixture was diluted and cooled before adding 100 mL of 40% sodium hydroxide. Next, a cylindrical beaker holding 50 mL of 4% boric acid was attached to the distillation apparatus, and the combination was evaporated into the acid. It was gathered once three droplets of indicator showed that the condensate amount was more significant than 150 mL. Next, ammonia was titrated with 0.1 M hydrochloric acid after being transformed into ammonium met borate. This method was used to determine the proportion of pure protein as (Eq. 6).

6 $${\text{Percentage }}\left( \% \right){\text{ of Protein}}\, = \,\frac{{{\text{titre~volume~sample}} - {\text{titre~volume~blank~x~}}0.014{\text{~x~}}0.1{\text{~x~}}6.25{\text{~}}}}{{{\text{weight~of~sample~used}}}} \times 100$$

#### Glycogen measurements

Anthrone reagent was used according to the procedure by [15] to measure glycogen stores in the liver and muscle. The following method was used in the determination of Glycogen as (Eq. 7):

7 $$\frac{{\text{DU}}}{{\text{DS}}}\,x\,0.1\,x\,\frac{\mathrm{volume \,of \,extract}}{\mathrm{ of \,tissue}}\, x \,100 \,x \,0.9\,=\,\mathrm{mg\, of \,Glycogen\, in }\,100\mathrm{ gm \,of\, tissue}$$

where:

DU indicates the unknown's optical density,

DS indicates the standard's optical density,

0.1 indicates the amount of glucose in (mg) in 2 ml of standard solution,

0.9 Which is the factor for converting glucose to Glycogen.

#### Total lipid measurements

The crude lipid content was extracted from the sample using a Soxhlet extractor with a mixture of chloroform and methanol (2:1, v/v). The crude lipid content was measured gravimetrically after oven-drying the extract at 80 °C overnight. The lipid content of the sample was then calculated using the formula (8) proposed by [13]:

8 $$Total \,Lipids\,\left( \% \right)\, = \,\frac{{{\text{weight of flask and extract fat}} - {\text{weight of empty flask }}}}{{\text{weight of dried sample}}} \times 100$$

### Nutritional value

The edible parts (muscles) of the studied species' caloric value was determined by applying the following formula based on the biochemical composition analysis provided by [20]:

9 $${\text{Nutritional value }}\left( {{\text{kcal}}/{1}00{\text{g}}} \right) \, = \, \left( {{\text{lipid}} \times { 9}} \right) \, + \, \left( {{\text{protein }} \times {4}} \right) \, + \, \left( {{\text{glycogen }} \times {4}} \right)$$

### Data analysis

The study utilized Principal Component Analysis (PCA) to examine the relationships between the chemical compositions of different fish species and metals via Bardawil lakes. PCA was used to reduce the dimensions of the observations and group similar ones together [46]. Additionally, Hierarchical Cluster Analysis was applied to fish species in the study area according to metal and chemical composition to identify similar groups of cases or variables. A one-way ANOVA analysis was also conducted to establish comparison efficiency [70]. All studies above were conducted using the R-4.2.1 program [33].

## Results and discussion

### Habitats and morphometric measurements of the studied fish

The investigated fish species are presented in Table (1), together with their average length, weight, health factor, environment, diet, and fishing importance. The fish showed an extensive and statistically significant range of variation in their body measurements. Species and size influence variation in total fish length, body weight, and condition factor.

### Heavy metals in fish

**Fig. 2:**
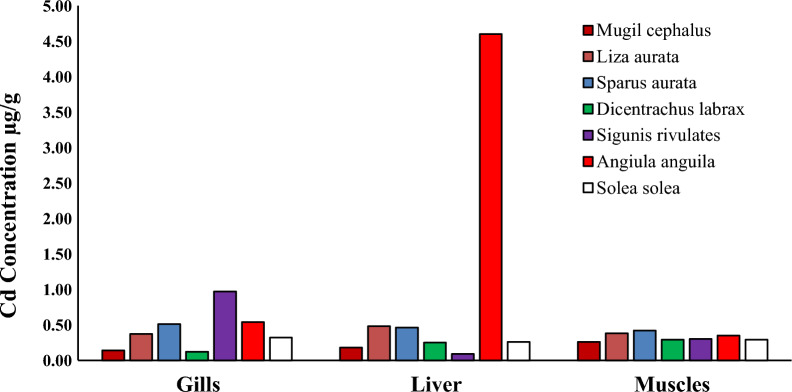
Cadmium concentration µg/g in different tissues in Bardawil Lake (area of investigation) during 2018

**Fig. 3:**
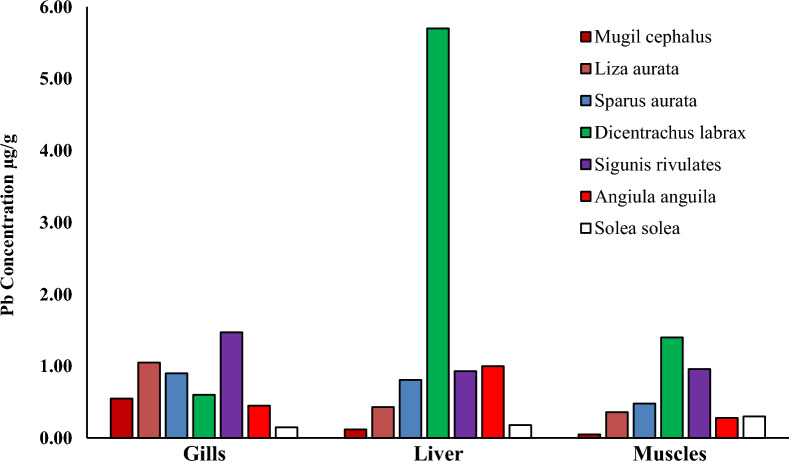
Lead concentration µg/g in different tissues in Bardawil Lake (area of investigation) during 2018

**Fig. 4:**
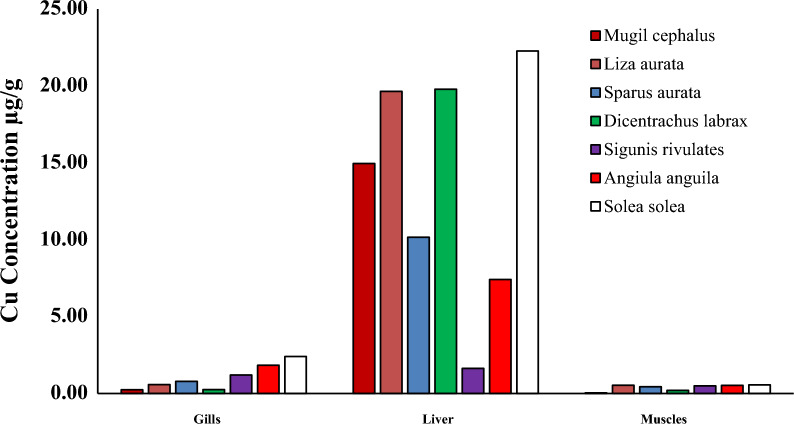
Copper concentration µg/ in different tissues in Bardawil Lake (area of investigation) during 2018

**Fig. 5:**
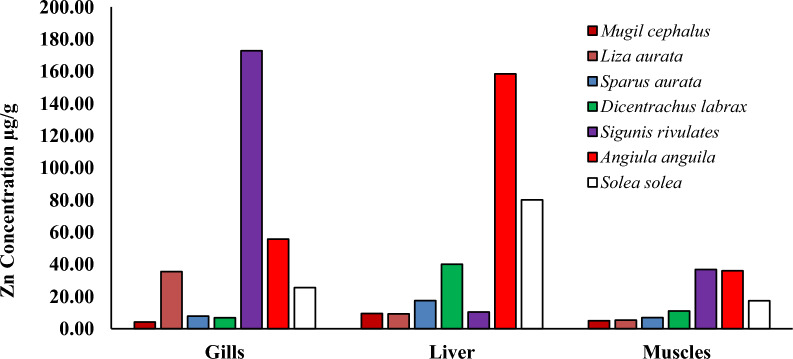
Zinc concentration µg/g in different tissues in Bardawil Lake (area of investigation) during 2018

**Fig. 6:**
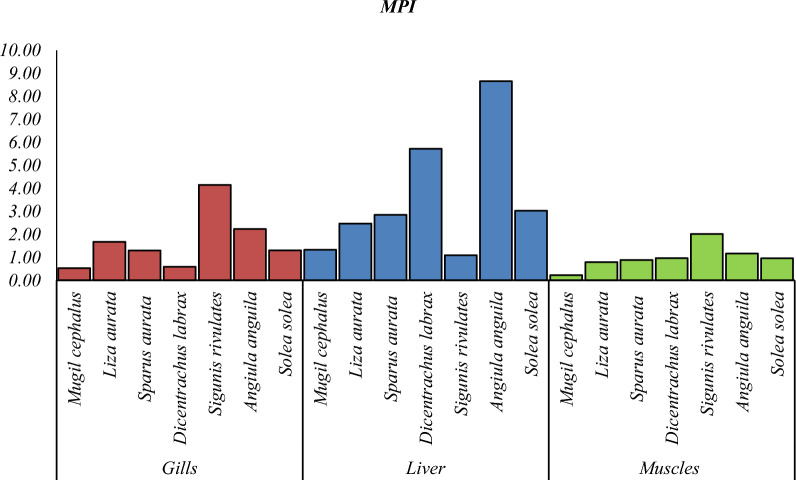
MPI concentration in different tissues in Bardawil Lake (area of investigation) during 2018

Heavy metal pollution is a significant environmental factor that can have a detrimental effect on the health of humans, as the consumption of fish muscles can result in serious health risks due to the bioaccumulation of heavy metals [3, 32]. Specific fish tissues acquire many heavy metals from natural and human sources [28, 59]. Researchers have examined economically important fish species to investigate their heavy metal content and nutritive value. The levels of (Cd, Pb, Cu and Zn) in the liver, gills, and muscles of seven species of fish (*Mugil cephalus, Liza auratus, Sparus aurata, Dicentrarchus labrax, Siganus rivulatus, Anguilla angilla, and Solae solea*) collected from Bardwil Lake, Egypt presented in Table 3 and Figs. 2, 3, 4, 5, 6. The amounts of metals in fish tissues often followed the order Zn > Cu > Pb > Cd. In addition, among the most studied fish species, Cd, Pb, Cu, and Zn concentrations were highest in the liver. The study found that the capability of tissues to collect metals varied in quantities. The liver recorded the highest values for Cd and Zn in *Angiula anguila*, whereas Pb and Cu in *Dicentrachus labrax and Solae solea*, respectively. Gills recorded the highest Cd, Pb, and Zn values in *Sigunis rivulates* and Cu in *Solae solea*. *Mugil cephalus* muscles recorded the lowest Cd, Pb, Cu, and Zn values. The concentrations of the metals in the tissues varied between (0.–4.60), (0.05–5.70), (0.04–22.27), and (4.08–172.75) µg/g wet weight for Cd, Pb, Cu, and Zn, respectively, in order of liver > gills > muscles. The highest levels of Cd, Pb, Cu, and Zn bioaccumulation are found in the liver and gill. Gill is the most efficient organ for cadmium detoxification, and the liver is the most efficient organ for copper [24]. The human population is mainly exposed to heavy metals via their consumption of fish and other aquatic animals, where the gills are often believed to be the most vulnerable organ, and muscle is the most widely consumed edible component [74]. The accumulation of heavy metals in fish tissues varied depending on the size, species, and habitat of the fish, as well as the level of pollution in the water. Moreover, it may be influenced by factors such as the fish's lifespan and physiological metabolism (Łuczyńska et al., 2020; [32]. Essential micronutrients such as Cu and Zn were more abundant in fish muscles than unnecessary and toxic elements Cd and Pb. The higher content of Cu and Zn in fish muscles may be attributed to the organism's automatic adsorption of these elements. Zn is crucial for functioning various body enzymes, while Cu is a component of various oxides [32]. Demersal fish, which are carnivores and feed primarily on shrimps, worms, and small benthic mollusks, had significantly higher levels of heavy metals than middle-upper fish [68], likely as a result of their greater exposure to silt, the primary source of heavy metals in oceanic fish. One possible explanation for the higher concentrations of heavy metals in demersal fish is that these fish consume many substrates rich in heavy metals, which promotes heavy metals transfer and accumulation in fish at different trophic levels by the food web [74].

**Table 3:** The liver and muscles` heavy metals concentration in different fish species from Bardawil Lake, Egypt

Organs	Fish Species	Heavy metals concentration µg/g (wet weight)				
		Cd	Pb	Cu	Zn	MPI
Muscles	*Mugil Cephalus*	0.26 ± 0.07^**d**^	0.05 ± 0.06^**e**^	0.04 ± 0.03^**g**^	4.91 ± 4.42^**e**^	0.22
	*Liza Auratus*	**0.38 ± 0.**09^b^	0.36 ± 0.12^**b**^	**0.53 ± 0.08**^**g**^	5.28 ± 4.32^**e**^	0.79
	*Sparus Aurata*	**0.42 ± 0.21**^**b**^	0.48 ± 0.25^**a**^	**0.44 ± 0.11**^**g**^	6.85 ± 2.50^**e**^	0.88
	*Dicentrarchus Labrax*	0.29 ± 0.13^**c**^	**1.00 ± 0.96**^**a**^	0.20 ± 0.21^**g**^	11.02 ± 7.21^**d**^	0.89
	*Siganus Rivulatus*	0.30 ± 0.33^**c**^	0.96 ± 0.30^**b**^	**0.50 ± 0.10**^**g**^	**36.81 ± 34.37**^**c**^	1.52
	*Anguilla Anguilla*	0.35 ± 0.32^**b**^	0.28 ± 0.17^**c**^	**0.52 ± 0.26**^**g**^	**36.01 ± 28.00**^**c**^	1.16
	*Solea Solea*	0.29 ± 0.17^**c**^	0.30 ± 0.30^**d**^	**0.56 ± 0.12**^**f**^	17.35 ± 12.10^**d**^	0.96
Liver	*Mugil Cephalus*	0.18 ± 0.06^**d**^	0.12 ± 0.00^**e**^	14.95 ± 17.33^**c**^	9.43 ± 3.48^**e**^	1.33
	*Liza Auratus*	0.48 ± 0.43^**b**^	0.43 ± 0.37^**c**^	19.64 ± 17.12^**b**^	9.17 ± 5.51^**e**^	2.47
	*Sparus Aurata*	0.46 ± 0.53^**b**^	0.81 ± 0.04^**a**^	10.16 ± 7.47^**d**^	17.42 ± 14.70^**d**^	2.84
	*Dicentrarchus Labrax*	0.25 ± 0.14^**d**^	**5.70 ± 5.24**^**a**^	18.78 ± 8.01^**b**^	40.08 ± 25.20^**c**^	5.72
	*Siganus Rivulatus*	0.09 ± 0.06^**f**^	0.93 ± 0.04^**a**^	1.63 ± 0.60^**f**^	10.34 ± 4.41^**e**^	1.09
	*Anguilla Anguilla*	**4.60 ± 4.11**^**a**^	1.04 ± 1.01^**b**^	7.41 ± 1.47^**e**^	**158.43 ± 200**^**a**^	8.66
	*Solea Solea*	0.26 ± 0.09^**d**^	0.18 ± 0.00^**e**^	**22.27 ± 4.38**^**a**^	80.05 ± 30.0^**b**^	3.03

-Values are expressed as (Mean ± SD.)

Heavy metal accumulation was most prominent in the liver and gills of fish. The unique structure of the gills allows for easy penetration of ions from water, making them the primary site for the direct absorption of heavy metals from the environment. In addition, heavy metal enrichment in the liver was associated with the induction and bonding of metallothionein, as the liver continuously accumulates, bio-transforms, and detoxifies heavy metals [28, 32, 63]. Results for fish muscles show that metals are within the allowable limits (FAO/WHO, 2015), while gills and liver are above the limits. Muscles have fewer binding proteins and enzyme processes, so they do not accumulate as much heavy metal as the liver. This study found that the estimated levels of all muscle metals were much lower than the dietary guidelines set by the United Nations Food and Agriculture Organization and the World Health Organization. (FAO/WHO, 2015).

Metal Pollution Index, MPI values varied from (0.22–8.56) (Table 3 and Fig. 6). MPI is a list of the fish in their muscles was reported: *Anguilla anguilla* > *Sigunis rivuletatus* > *Solae solea* > *Dicentrachus labrax* > *Sparus aurata* > *Liza auratus* > *Mugil cephalus* > *Sigunis rivuletatus*. The liver got the highest MPI value among the organs we studied, while the gills and muscles scored the lowest. However, the distribution of metals in fish changes depending on the route of exposure. Metals may be absorbed by fish either via their diet or by contact with polluted water on their respiratory surfaces [74].

### Health risk assessment

#### Non-carcinogenic hazard (THQ)

This study's findings that THQ values for normally consumed fish tissues from Bardawil Lake were below 1 imply that these fish pose no known health hazards to humans. Whereas, THQ values for high-consuming fish tissues were > 1, especially muscles for Cd in (*Liza auratus* and *Sparus aurata*) and Pb in (*Dicentrachus labrax* and *Sigunis rivulates*), indicating a likely adverse health effect from heavy metal exposure (Table 4). Consuming fish muscles from Bardawil Lake has no public health risk, but high-consumption individuals may have adverse consequences from ingesting Cd and Pb found in certain species. Predicting the probable impacts of pollutants on people requires considering the additive effect of contaminants on the population for non-carcinogenic risk [35, 74]. According to USEPA [66] recommendations, a THQ value of < 1 indicates no risk to human health or no damaging effects of heavy metals on human health from consuming fish daily.

**Table 4:** Target hazard quotient (THQ) and hazard index (HI) of heavy metals in the fish tissues consumed by normal and high consumers from Bardawil Lake (* is the reference dose of each metal)

Tissue		Fish Species	Heavy metals concentration µg/g (wet weight)				
			THQ_Cd_	THQ_Pb_	THQ_Cu_	THQ_Zn_	HI (TTHQ)
Normal Consumer	Muscles	*Mugil Cephalus*	0.13	0.01	0.01	0.01	0.26
		*Liza Auratus*	0.34	0.08	0.01	0.11	0.46
		*Sparus Aurata*	0.47	0.11	0.02	0.02	0.52
		*Dicentrarchus Labrax*	0.11	0.32	0.01	0.02	0.63
		*Siganus Rivulatus*	0.89	0.68	0.03	0.53	**1.08**
		*Anguilla Anguilla*	0.50	0.06	0.04	0.17	0.50
		*Solea Solea*	0.29	0.07	0.06	0.08	0.40
	Liver	*Mugil Cephalus*	0.17	0.03	0.34	0.03	0.57
		*Liza Auratus*	0.44	0.10	0.45	0.03	**1.01**
		*Sparus Aurata*	0.42	0.19	0.23	0.05	0.89
		*Dicentrarchus Labrax*	0.23	1.30	0.43	0.12	2.08
		*Siganus Rivulatus*	0.08	0.21	0.04	0.03	0.36
		*Anguilla Anguilla*	4.23	0.24	0.17	0.49	5.10
		*Solea Solea*	0.24	0.04	0.51	0.25	**1.03**
	Gills	*Mugil Cephalus*	0.24	0.13	0.00	0.02	0.27
		*Liza Auratus*	0.35	0.24	0.01	0.02	0.70
		*Sparus Aurata*	0.39	0.21	0.01	0.02	0.71
		*Dicentrarchus Labrax*	0.27	0.14	0.00	0.03	0.27
		*Siganus Rivulatus*	0.28	0.34	0.01	0.11	**1.78**
		*Anguilla Anguilla*	0.32	0.10	0.01	0.11	0.81
		*Solea Solea*	0.26	0.03	0.01	0.05	0.46
Higher Consumer	Muscles	*Mugil Cephalus*	0.73	0.04	0.00	0.05	0.82
		*Liza Auratus*	1.09	0.26	0.04	0.05	1.44
		*Sparus Aurata*	1.20	0.34	0.03	0.07	1.64
		*Dicentrarchus Labrax*	0.84	1.00	0.01	0.10	1.95
		*Siganus Rivulatus*	0.86	2.11	0.04	0.35	3.36
		*Anguilla Anguilla*	0.99	0.20	0.04	0.34	1.57
		*Solea Solea*	0.82	0.21	0.04	0.17	1.24
	Liver	*Mugil Cephalus*	0.53	0.09	1.07	0.09	1.77
		*Liza Auratus*	1.36	0.31	1.40	0.09	3.16
		*Sparus Aurata*	1.30	0.58	0.73	0.17	2.78
		*Dicentrarchus Labrax*	0.71	4.07	1.34	0.38	6.51
	Gills	*Siganus Rivulatus*	0.26	0.66	0.12	0.10	1.14
		*Anguilla Anguilla*	13.15	0.74	0.53	1.51	15.93
		*Solea Solea*	0.75	0.13	1.59	0.76	3.23
		*Mugil Cephalus*	0.41	0.39	0.02	0.04	0.85
		*Liza Auratus*	1.05	0.75	0.04	0.34	2.18
		*Sparus Aurata*	1.45	0.64	0.06	0.07	2.23
		*Dicentrarchus Labrax*	0.34	0.43	0.02	0.06	0.85
		*Siganus Rivulatus*	2.78	1.05	0.09	1.65	5.56
		*Anguilla Anguilla*	1.55	0.32	0.13	0.53	2.54
		*Solea Solea*	0.90	0.11	0.17	0.24	1.43
	* TDI_s_ (µg/day)* (WHO,1989)*		58.3*	105*	700*	8000–11000*	

^*^ Toxicological limit (µg/day) (FAO/WHO, 1989)

#### Carcinogenic risk

Cancer risk was computed using each metal's relevant cancer slope factors, and the results are reported in Table 5; carcinogenicity and other exposure characteristics were also used to estimate health hazards associated with fish consumption [35]. Except for Cd, which was more significant in several species in both normal and high consumers, the fish intake from Bardawil Lake was within USEPA guidelines. From 1.0 × 10^–6^ to 1.0 × 10^–4^, the US Environmental Protection Agency (USEPA) has set its cancer risk standards [66].

**Table 5:** Carcinogenic risk assessment of heavy metals in the fish tissues consumed by normal and high consumers from Bardawil Lake during, 2018

Organs		Fish Species	Cd	Pb	Cancer Slop
**Normal Consumer**	**Muscles**	*Mugil Cephalus*	8.1E-05	8.9E-08	8.2E-05
		*Liza Auratus*	1.2E-04	6.4E-07	1.2E-04
		*Sparus Aurata*	1.3E-04	8.5E-07	1.3E-04
		*Dicentrarchus Labrax*	9.3E-05	2.5E-06	9.5E-05
		*Siganus Rivulatus*	9.6E-05	5.3E-06	1.0E-04
		*Anguilla Anguilla*	1.1E-04	5.0E-07	1.1E-04
		*Solea Solea*	9.1E-05	5.3E-07	9.1E-05
	**Liver**	*Mugil Cephalus*	5.8E-05	2.1E-07	5.9E-05
		*Liza Auratus*	1.5E-04	7.6E-07	1.5E-04
		*Sparus Aurata*	1.5E-04	1.4E-06	1.5E-04
		*Dicentrarchus Labrax*	7.9E-05	1.0E-05	8.9E-05
		*Siganus Rivulatus*	2.9E-05	1.7E-06	3.0E-05
		*Anguilla Anguilla*	1.5E-03	1.8E-06	1.5E-03
		*Solea Solea*	8.3E-05	3.2E-07	8.3E-05
	**Gills**	*Mugil Cephalus*	4.5E-05	9.8E-07	4.6E-05
		*Chelon Auratus*	1.2E-04	1.9E-06	1.2E-04
		*Sparus Aurata*	1.6E-04	1.6E-06	1.6E-04
		*Dicentrarchus Labrax*	3.8E-05	1.1E-06	3.9E-05
		*Siganus Rivulatus*	3.1E-04	2.6E-06	3.1E-04
		*Anguilla Anguilla*	1.7E-04	8.0E-07	1.7E-04
		*Solea Solea*	1.0E-04	2.7E-07	1.0E-04
High Consumer	Muscles	*Mugil Cephalus*	2.5E-04	2.8E-07	2.5E-04
		*Liza Auratus*	3.8E-04	2.0E-06	3.8E-04
		*Sparus Aurata*	4.2E-04	2.7E-06	4.2E-04
		*Dicentrarchus Labrax*	2.9E-04	7.8E-06	3.0E-04
		*Siganus Rivulatus*	3.0E-04	1.6E-05	3.2E-04
		*Anguilla Anguilla*	3.5E-04	1.6E-06	3.5E-04
		*Solea Solea*	2.8E-04	1.7E-06	2.9E-04
	Liver	*Mugil Cephalus*	1.8E-04	6.7E-07	1.8E-04
		*Liza Auratus*	4.7E-04	2.4E-06	4.8E-04
		*Sparus Aurata*	4.5E-04	4.5E-06	4.6E-04
		*Dicentrarchus Labrax*	2.5E-04	3.2E-05	2.8E-04
		*Siganus Rivulatus*	9.0E-05	5.2E-06	9.5E-05
		*Anguilla Anguilla*	4.6E-03	5.8E-06	4.6E-03
		*Solea Solea*	2.6E-04	1.0E-06	2.6E-04
	Gills	*Mugil Cephalus*	1.4E-04	3.1E-06	1.4E-04
		*Liza Auratus*	3.6E-04	5.8E-06	3.7E-04
		*Sparus Aurata*	5.1E-04	5.0E-06	5.1E-04
		*Dicentrarchus Labrax*	1.2E-04	3.3E-06	1.2E-04
		*Siganus Rivulatus*	9.7E-04	8.2E-06	9.7E-04
		*Anguilla Anguilla*	5.4E-04	2.5E-06	5.4E-04
		*Solea Solea*	3.1E-04	8.3E-07	3.1E-04

Limits = 1.0 × 10^–6^ to 1.0 × 10^–4^ [66]

#### The combined risk of many metals (HI)

**Fig. 7:**
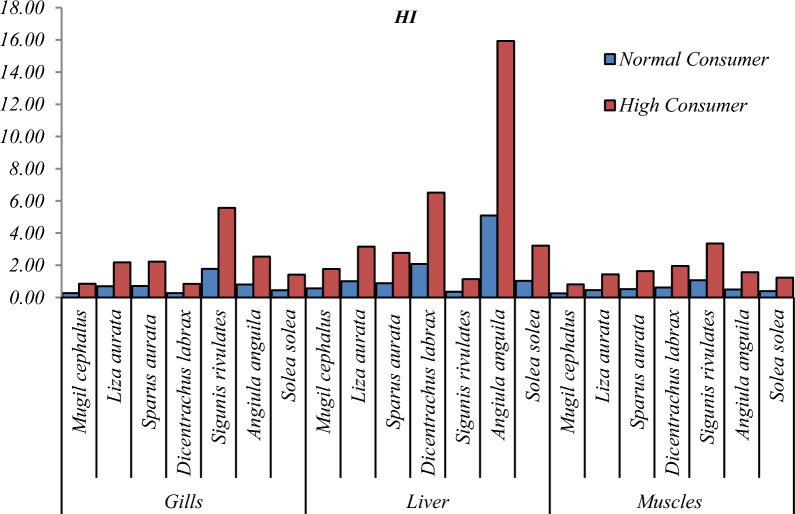
HI concentration in different tissues for normal and high consumers in Bardawil Lake (area of investigation) during 2018

Except for a few species, the HI values for normal consumption of all tested fish muscle species were < 1, indicating that none were detrimental to the body at the usual ingestion rate for a healthy adult. Although Cd and Pb are only found in low concentrations in the body, their presence has been linked to a variety of health problems [19, 35] (Table 4, Fig. 7). THQ < 1, as recommended by USEPA [66], poses no threat to human health. HI values were > 1 in normal and high consumers, ranging from 0.27 to 5.10 and 0.85 to 15.93, respectively. This study's results corroborated those published by Ezemonye et al. [19], indicating that eating fish has no health hazards since THQ was > 1.

### Fish biochemical composition

Fish species that have been studied contain abundant levels of crude protein, lipids, Moisture, and ash, meeting human nutritional requirements. Protein is the most expensive, crucial part of a fish's diet and plays a significant role in energy balance by creating ATP [6, 50].

Lipids are the fish's primary non-protein energy source and contain essential fatty acids that contribute to growth and health [6]. By including an adequate amount of lipids in fish diets, catabolism of dietary protein can be reduced and used for energy purposes [27]. Fish possess Glycogen as a reserve of carbohydrates in their liver, which can be converted to glucose with the help of specific enzymes. Certain carbohydrates in fish are transformed into lipids and stored in muscles and the liver. Glucose can participate in fish's energy supply and metabolic activities by converting from Glycogen if necessary. The amount of Glycogen present in muscle, especially the liver, is believed to indicate the body's physiological state [44].

**Fig. 8:**
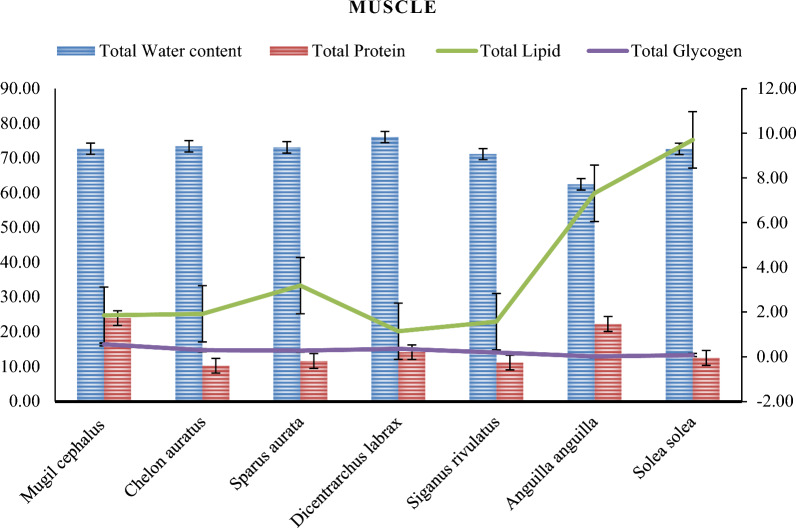
The muscle's biochemical composition in different fish species from Bardawil Lake (area of investigation) during 2018

**Fig. 9:**
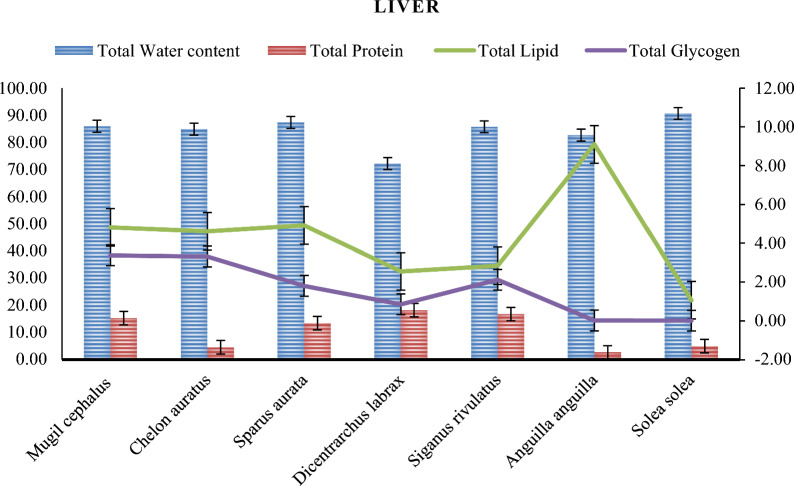
The liver's biochemical composition in different fish species from from Bardawil Lake (area of investigation) during 2018

Tables 6, 7 and Figures 8, 9 display the biochemical composition of fish muscles and liver. The differences in the biochemical composition of marine fish investigated in this study can be attributed to species, food availability, and geographical location [4]. All results demonstrate significant differences between various species (*P*< 0.05).

**Table 6:** The muscles and liver Bio-chemical composition in different fish species from Bardawil Lake, Egypt

Tissue	Fish Species	Biochemical Composition %			
		Protein*	Lipid*	Glycogen*	Moisture**
Muscles	*Mugil Cephalus*	23.96 ± 4.27^**c**^	1.86 ± 0.87^**g**^	0.55 ± 0.16^**e**^	72.70 ± 1.41^**c**^
	*Liza auratus*	10.26 ± 1.99^**i**^	1.92 ± 0.59^**g**^	0.29 ± 0.23^**f**^	73.40 ± 1.17^**c**^
	*Sparus Aurata*	11.62 ± 2.47^**e**^	3.19 ± 1.02^**e**^	0.27 ± 0.05^**f**^	73.08 ± 1.19^**c**^
	*Dicentrarchus Labrax*	14.15 ± 1.10^**b**^	1.14 ± 0.53^**h**^	0.35 ± 0.04^**e**^	76.03 ± 1.29^**c**^
	*Siganus Rivulatus*	11.17 ± 1.21^**b**^	1.57 ± 0.57^**g**^	0.19 ± 0.07^**f**^	71.13 ± 1.21^**c**^
	*Anguilla Anguilla*	22.29 ± 2.31^**j**^	7.31 ± 5.10^**c**^	0.17 ± 0.00^**f**^	70.00 ± 2.65^**c**^
	*Solea Solea*	12.50 ± 1.55^**i**^	9.70 ± 4.78^**a**^	0.18 ± 0.06^**f**^	72.62 ± 2.53^**c**^
Liver	*Mugil Cephalus*	15.29 ± 6.52^**a**^	4.82 ± 0.21^**d**^	3.38 ± 0.01^**a**^	85.90 ± 1.27^**a**^
	*Liza auratus*	4.56 ± 3.04^**h**^	4.61 ± 0.39^**d**^	3.32 ± 0.88^**a**^	84.82 ± 10.11^**a**^
	*Sparus Aurata*	13.38 ± 3.08^**g**^	4.92 ± 0.10^**d**^	1.80 ± 1.18^**c**^	87.35 ± 1.47^**a**^
	*Dicentrarchus Labrax*	18.19 ± 2.95^**d**^	2.55 ± 0.14^**f**^	0.85 ± 0.25^**d**^	72.20 ± 1.01^**c**^
	*Siganus Rivulatus*	16.79 ± 3.72^**g**^	2.84 ± 0.05^**e**^	2.12 ± 0.57^**b**^	85.70 ± 1.59^**a**^
	*Anguilla Anguilla*	2.75 ± 0.87^**a**^	9.10 ± 4.86^**b**^	0.32 ± 0.01^**f**^	82.65 ± 2.41^**b**^
	*Solea Solea*	4.99 ± 5.14^**f**^	1.06 ± 0.49^**i**^	0.23 ± 0.00^**f**^	90.66 ± 7.29^**a**^

-Values are expressed as (Mean ± SD.)

- Different letters following the means in each column are significantly different (P ≤ 0.05)

^*^Values are expressed as (%) of the dry weight;

^**^Values are expressed as (%) of the fresh weigh

**Table 7:** The muscles and live Caloric value in different fish species from Bardawil Lake, Egypt

Tissue	Fish Species	Total Protein	Total Lipid	Total Glycogen	Caloric value (kcal/100 g
Muscles	*Mugil Cephalus*	95.84	16.70	2.21	114.75
	*Liza Auratus*	41.03	17.28	1.18	59.48
	*Sparus Aurata*	46.48	28.69	1.09	76.26
	*Dicentrarchus Labrax*	56.61	10.26	1.41	68.28
	*Siganus Rivulatus*	44.66	14.15	0.76	59.57
	*Anguilla Anguilla*	89.14	65.80	0.05	155.62
	*Solea Solea*	50.00	87.33	0.29	138.05
Liver	*Mugil Cephalus*	61.16	43.34	13.52	118.02
	*Liza Auratus*	18.22	41.49	13.27	72.98
	*Sparus Aurata*	53.54	44.24	7.21	105.00
	*Dicentrarchus Labrax*	72.76	22.95	3.41	99.12
	*Siganus Rivulatus*	67.14	25.59	8.47	101.20
	*Anguilla Anguilla*	11.00	81.87	0.06	94.15
	*Solea Solea*	19.96	9.54	0.03	30.42

**-**Values are expressed as (Mean ± SD.)

- Different letters following the means in each column are significantly different (P ≤ 0.05)

#### Fish biochemical composition

Table 6 and Figs. 8, 9 show the muscle and liver moisture content, respectively. The livers of most fish species exhibited higher biochemical composition values, including protein, lipids, Glycogen, and water content, than muscles. The moisture content of the studied fish muscles ranged from 70.00 ± 2.65% to 76.03 ± 1.29%. *Dicentrarchus labrax* had the highest moisture content, followed by *Liza auratus* and *Sparus aurata*, while *Anguilla anguilla* had the lowest. In contrast, Moisture content in the liver ranges from 72.20 ± 1.01 to 90.66 ± 7.29, with the highest percentage in *Solae solea*, followed by *Sparus aurata, Mugil cephalus,* and *Siganus rivulatus,* and the lowest in *Dicentrarchus labrax.* Moisture content in fish muscles is influenced by factors such as season, age, and environment, which could explain the observed differences in moisture content between species [32].

The crude protein content in fish muscles ranged from 10.26 ± 1.99% to 23.96 ± 4.27%, with *Mugil cephalus* having the highest protein content, followed by *Anguilla anguilla, and Liza auratus* having the lowest. Protein content in the liver (%) ranged from 2.75 ± 0.87 to 18.19 ± 2.95, with the highest percentage in *Dicentrarchus labrax*, followed by *S. Rivulatus* and *Mugil cephalus*. The lowest percentage was found in *Anguilla anguilla* and *Solae solea*. These results could be attributed to differences in feeding habits, ecological conditions, and food availability [72]. Consistent with other research, we discovered no association between protein abundance and heavy metal concentrations in fish muscle (Pearson correlation test, P < 0.05).

The crude lipid content in fish muscles ranged from 1.14 ± 0.53% to 9.70 ± 4.78%, with *Solae solea* having the highest lipid content and *Dicentrarchus labrax* having the lowest. Whereas the lipid content in the liver (%) ranged from 9.10 ± 4.86 to 1.06 ± 0.49, with the highest level observed in *Anguilla anguilla* and the lowest in *Solae solea* (1.06 ± 0.49), followed by *Dicentrarchus labrax* (2.55 ± 0.14) and *Siganus rivulatus* (2.84 ± 0.05). The fish's liver contains high levels of total protein, which may be necessary for generative metabolism and spawning processes. The fish's ability to utilize dietary lipids as a non-protein energy source is influenced by the content of dietary carbohydrates, which can also serve as non-protein energy sources [26]. Similar to the protein content results, no correlation was observed between lipid content and heavy metals in fish muscle (*P* < 0.05). Moreover, lipid content is affected by factors such as environment, life cycle, and topographical origin [72].

The glycogen content in fish muscles ranged from 0.17 ± 0.06% to 0.55 ± 0.16%, with *Mugil cephalus* having the highest values and *Dicentrarchus labrax* and *Anguilla anguilla* having the lowest. Meanwhile, glycogen content in the liver (%) ranged from 0.23 ± 0.00%—3.38 ± 0.01%, with the highest levels observed in *Mugil cephalus* and *Liza auratus*, while the lowest levels were found in *Solae solea* and *Anguilla anguilla*. The utilization of Glycogen and lipids for energy metabolism in fish are closely interrelated. Fish can use products of fat metabolism and lipids from carbohydrate metabolism to produce Glycogen, which may vary due to physiological processes during the pre-spawning period and environmental factors that affect the organism's functional activity [38, 61, 62]. These factors could influence the differences observed in fish, as noted by studies conducted by [48, 51]. Therefore, the differences in their levels may be related to the physiological processes during the pre-spawning period and influenced by environmental factors that affect the organism's functional activity. The difference in the number of biochemical compounds in fish muscle could depend on various factors, such as the quality of water, type of feeding, time of capture, fish life cycle, and farming system [54].

One of the most important indicators of the fish's physiological status is the metabolic process, which is based on markers of metabolic processes linked to the biosynthesis of proteins, lipids, carbohydrates, and other organic compounds that support the body's adaptation to altered ecological conditions of existence [39, 60]. Metabolic indicators are used to assess the physiological state of fish and the ecological status of the water bodies they inhabit [52, 55].

Heavy metals can interact with the biochemical composition of living organisms, including fish, leading to changes in their physiological processes and functions. Heavy metals can bind to proteins, enzymes, and DNA, leading to changes in their conformation, activity, and stability, ultimately affecting the organism's health and survival [10].

Heavy metals can also disrupt the metabolism of essential components such as lipids, carbohydrates, and amino acids in living organisms, including fish, as noted by [29, 31].

## Data analysis

The article presents Table 3 and 6, which discuss ANOVA analysis with multiple comparison analyses of the nutritional values for the liver and muscle of various fish species and their metal concentrations. A*nguilla anguilla* and *Solae solea* contain the lowest concentrations of cadmium, copper, and zinc among the species. Regarding nutritional values, *Mugil cephalus* and *Anguilla anguilla* have significantly high protein levels, while *Solae solea* has the highest value of lipids. In addition, *Mugil cephalus* and *Liza auratus* have significantly higher levels of Glycogen. Meanwhile, *Mugil cephalus*, *Liza auratus*, *Sparus aurata*, *S, Siganus rivulatus*, and *Solae solea* have the highest moisture content.

**Fig. 10:**
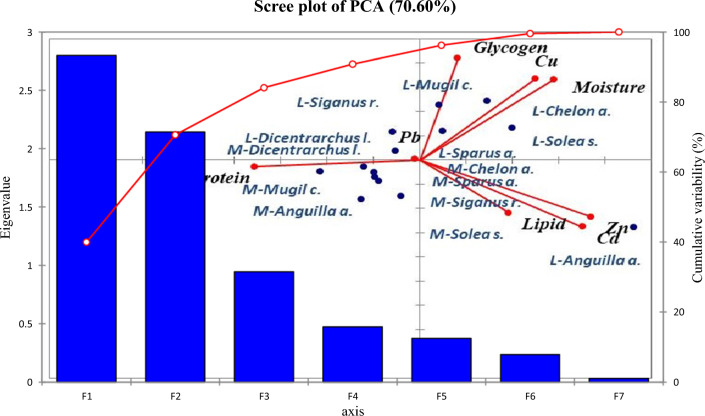
Principal component analysis of metal and chemical composition for different fish species in Bardawil Lake

The variability of the nutritional values with metal concentrations in Bardawil Lake was analyzed using principal component analysis, and it showed a variability of 70.60%. Factors one and two had 2.79 and 2.14, respectively, with eigenvalues less than one being recorded. As illustrated in (Fig. 10), *Anguilla anguilla* showed the highest correlation with zinc, cadmium, and protein. Copper, Glycogen, and moisture content closely influenced *Mugil cephalus*, *Liza auratus*, *Sparus aurata*, and *Solae solea*.

**Fig. 11:**
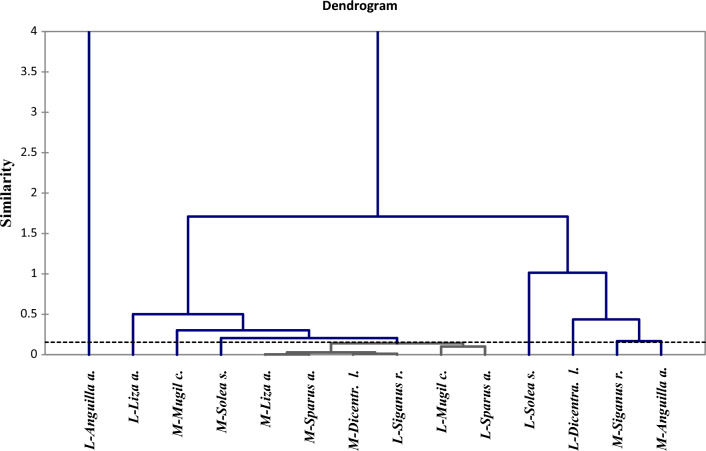
Hierarchical Cluster Analysis for the liver nutritional value of different fish species from Bardawil Lake (area of investigation) during 2018

The dendrogram in (Fig. 11) displays a hierarchical cluster analysis technique of fish species in different lakes based on their liver and muscle nutritional values. It reveals that 14 data points were linked and grouped into eight main categories based on conventional Euclidean distance. Muscles for *Anguilla anguilla* and *Siganus rivulatus* are strongly correlated with other species. Moreover, the muscles of *Liza auratus*, *Sparus aurata*, *Dicentrarchus labrax* and the liver of *Mugil cephalus*, *Sparus aurata*, and *Siganus rivulatus* are closely related. Meanwhile, liver *Anguilla anguilla* was isolated in a single dendrogram branch.

## Conclusion

Fish caught from Bardawil Lake (*Mugil cephalus, Liza auratus, Sparus aurata, Dicentrarchus labrax, Siganus rivulatus, Anguilla anguilla, and Solae solea*) are a great source of protein and fat. Still, there is some concern that they may pose a health risk to consumers. Due to their eating habits and environmental preferences, the liver tissues of demersal fish species contain greater concentrations of metals than those of pelagic fish species. Using MPI and HI values, we determined the metal load of edible fish tissues produced by different human acts. At the same time, THQ and HI levels were > 1.0, especially in the gills and liver for higher consumer fish tissues. The proposed human risk assessment considers dose and consumption-dependent parameters to estimate human consumers' dangers better. Heavy metals may poison humans; therefore, we must enforce strict control methods to keep them below acceptable proportions in the shellfish we consume. Metal evaluations and warnings for seafood consumption should be routinely performed in bodies of water near densely populated areas.

## Data Availability

The raw data supporting the conclusions of this manuscript would be available by the authors, without undue reservation, to any qualified researcher.
